# Diffuse large B‐cell lymphoma‐induced intussusception: A case report and literature review

**DOI:** 10.1002/ccr3.9046

**Published:** 2024-06-18

**Authors:** Hao‐Cheng Chang, Jung‐Cheng Kang, Ta‐Wei Pu, Ruei‐Yu Su, Chao‐Young Chen, Je‐Ming Hu

**Affiliations:** ^1^ Department of Surgery, Tri‐Service General Hospital National Defense Medical Center Taipei Taiwan; ^2^ Department of Surgery, Division of Colon and Rectal Surgery Taiwan Adventist Hospital Taipei Taiwan; ^3^ Division of Colon and Rectal Surgery, Department of Surgery, Tri‐Service General Hospital Songshan Branch National Defense Medical Center Taipei Taiwan; ^4^ Division of Colon and Rectal Surgery, Department of Surgery, Tri‐Service General Hospital National Defense Medical Center Taipei Taiwan; ^5^ Department of Pathology, Tri‐Service General Hospital National Defense Medical Center Taipei Taiwan; ^6^ Department of Pathology and Laboratory Medicine Taoyuan Armed Forces General Hospital Taoyuan Taiwan

**Keywords:** adult intussusception, diffuse large B‐cell lymphoma, immunohistochemical markers, laparoscopic right hemicolectomy

## Abstract

Adult intussusception necessitates early surgical intervention. We emphasis the significance of considering diffuse large B‐Cell lymphoma in differential diagnoses for adult intussusception, particularly in the colon, to ensure precise diagnosis and optimal management.

## 1 INTRODUCTION

Adult intussusception, an uncommon yet critical gastrointestinal (Gl) event, is frequently a sentinel of underlying malignancies.^1^, ^2^ The subtle and non‐specific clinical manifestations of the condition contrast starkly with its potential to precipitate severe clinical outcomes, making precise diagnosis and swift management indispensable. While intussusception is largely perceived as a pediatric ailment, its adult form (accounting for a mere 5% of all cases and 1%–5% of intestinal obstructions^3^) demands a nuanced understanding of its epidemiology and pathophysiology to enhance patient care.

In the realm of adult Gl pathologies, intussusception represents a diagnostic conundrum, with approximately 90% of cases attributable to identifiable lead points ranging from benign polyps to aggressive malignancies such as diffuse large B‐Cell lymphoma (DLBCL), which alone is implicated in 65%–75% of adult occurrences.^2^, ^4^ This case report amalgamates two distinct narratives of DLBCL‐associated intussusception, converging on a common goal: to refine the existing diagnostic protocols and therapeutic approaches within this domain. By dissecting the clinical journey from symptom onset to the intervention, we emphasize the critical role of advanced imaging modalities and of surgery, not just as therapeutic necessities but also as prognostic determinants in adult intussusception secondary to DLBCL.

Our integrative analysis underscores the urgent need for a sophisticated clinical algorithm that puts emphasis in early recognition, comprehensive imaging, and surgical resolution to address the etiological complexity of this condition. The synthesis of our case studies aims to fill the void in evidence‐based guidelines for adult intussusception, steering the discourse towards a more proactive and patient‐focused paradigm in oncological surgical practice.

## 2 CASE HISTORY/ EXAMINATION

A 71‐year‐old multiparous nonsmoker woman sought medical attention in our outpatient department, reporting intermittent right lower abdominal pain. This symptomatology was concomitant with an unintentional weight loss of approximately 6 kg over 2 months. The patient had no history of abdominal surgical intervention but had well‐managed hypertension and Parkinson's disease, both effectively controlled with medication.

Upon thorough physical examination, the patient exhibited pallor, but her body temperature and vital signs remained within normal ranges. In the right lower quadrant of the abdomen, palpation revealed a tender mass with a regular border, measuring 5–6 cm in diameter. It was soft to the touch yet immobile, suggesting possible attachment to deeper structures, with mild tenderness noted upon examination.

Hematological assessments revealed a white blood cell count of 7.61 × 10^3^/μL, comprising 67.8% neutrophils and 24.0% lymphocytes. C‐reactive protein (CRP) levels were quantified as 0.89 mg/dL.

**FIGURE 1:**
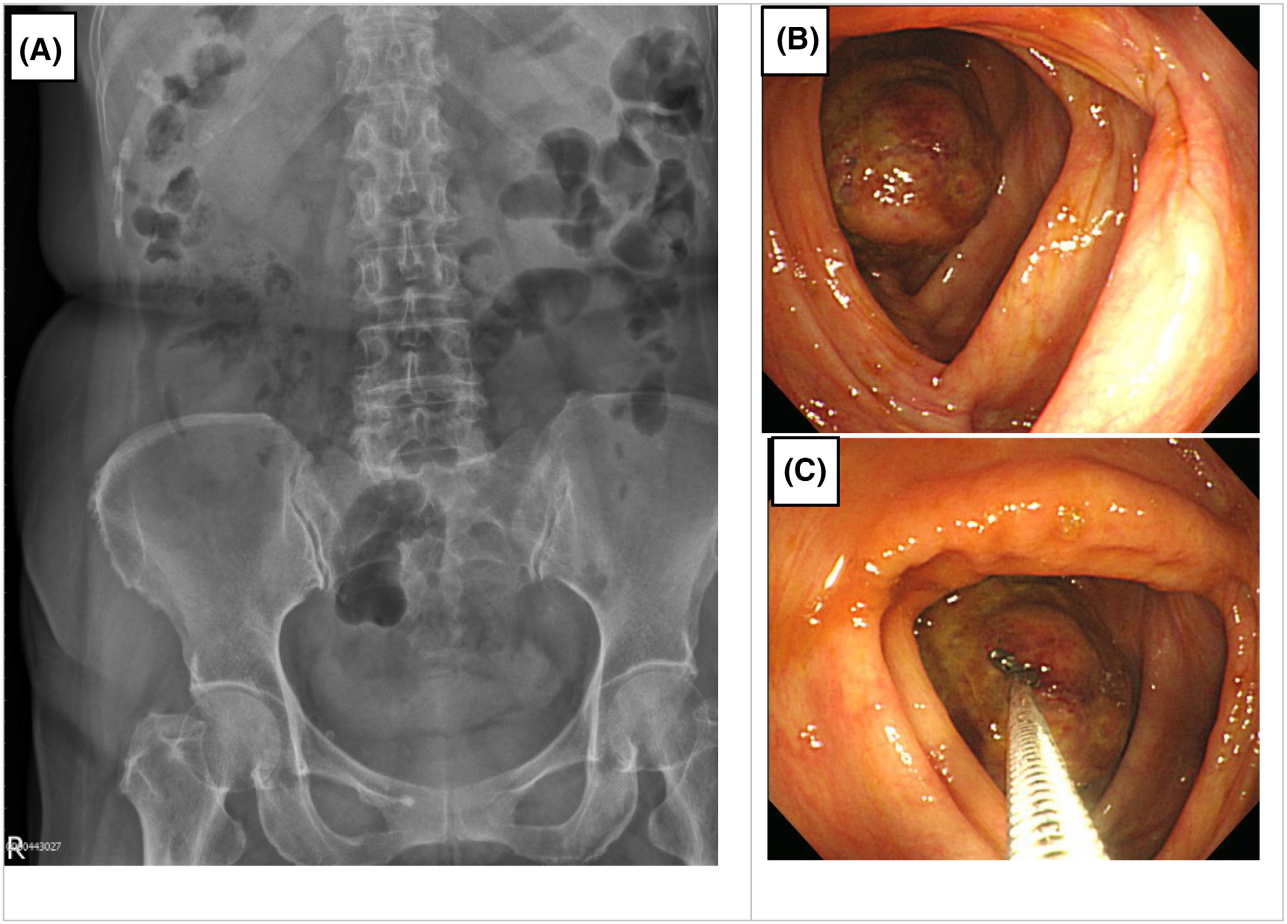
(A) Abdominal X‐ray: The X‐ray of the abdomen exhibited signs of mild to moderate ileus without definitive evidence of mechanical obstruction. (B) Colonoscopic Image: A colonoscopic examination revealed the presence of a ball‐like mass with an irregular surface. (C) Colonoscopic Image: The colonoscopy also enabled the acquisition of biopsies for further analysis.

Following the initial assessment, hepatic and renal function tests were conducted, with results within the normal range. Furthermore, serum levels of carcinoembryonic antigen (CEA) and carbohydrate antigen 19–9 (CA 19‐9) were within the normal reference ranges. After these evaluations, an abdominal x‐ray was performed, revealing unremarkable findings. A total colonoscopy identified an irregular, ball‐like mass causing complete luminal obstruction. Biopsies were meticulously obtained from affected areas for further analysis (refer to Figure 1 for detailed illustrations). A chest radiograph demonstrated the absence of pulmonary tumors. Histopathological examination of the biopsy specimen revealed replete ulceration with necrotic debris, accompanied by active nonspecific cellular infiltration, predominantly neutrophils and round cells.

Following the initial assessments, an abdominal computed tomography (CT) scan revealed a heterogeneously enhancing lesion measuring approximately 2.8 × 3.4 cm in the proximal ascending colon. Smaller nodules were also detected within the mesentery of the cecal region, raising suspicion of metastatic lymphadenopathy.

## 3 METHODS (DIFFERENTIAL DIAGNOSIS, INVESTIGATIONS AND TREATMENT)

**FIGURE 2:**
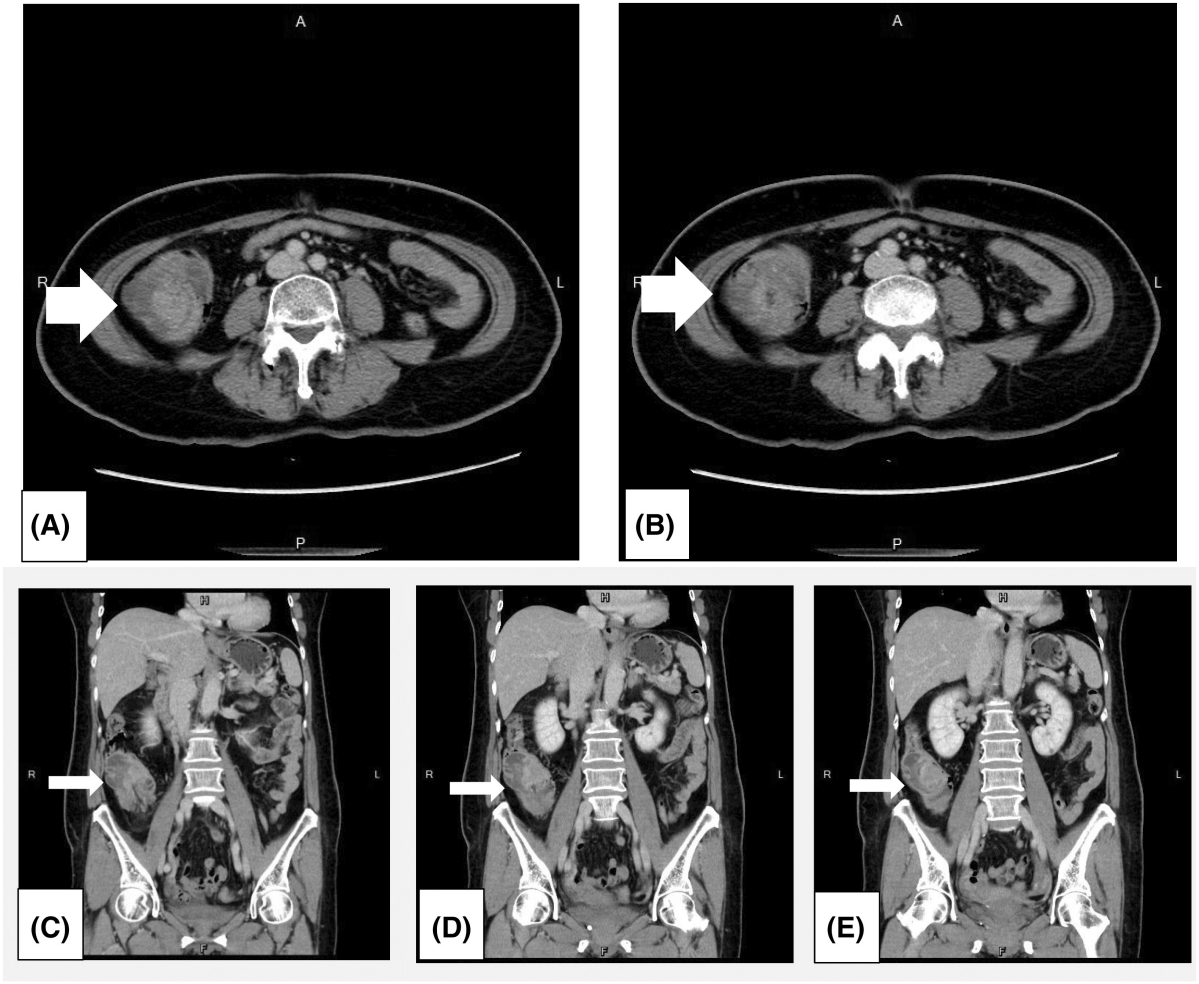
Abdominal Computed Tomography (CT) scans provided multiple perspectives of the intussusception: (A) Axial View: The axial view displayed the classic ‘target sign’, which is characteristic of ileocolic intussusception. (B) Reniform Mass Lesion: A reniform mass lesion was observed, attributed to edematous changes and increased mural thickness. (C, D, & E) Coronal Views: Coronal views provided additional confirmation of ileocecal intussusception, as indicated by the directional arrows.

These findings led to a diagnosis of ileocolic intussusception with suspected tumor involvement, necessitating laparoscopic right hemicolectomy (refer to Figure 2 for detailed visual representations).

**FIGURE 3:**
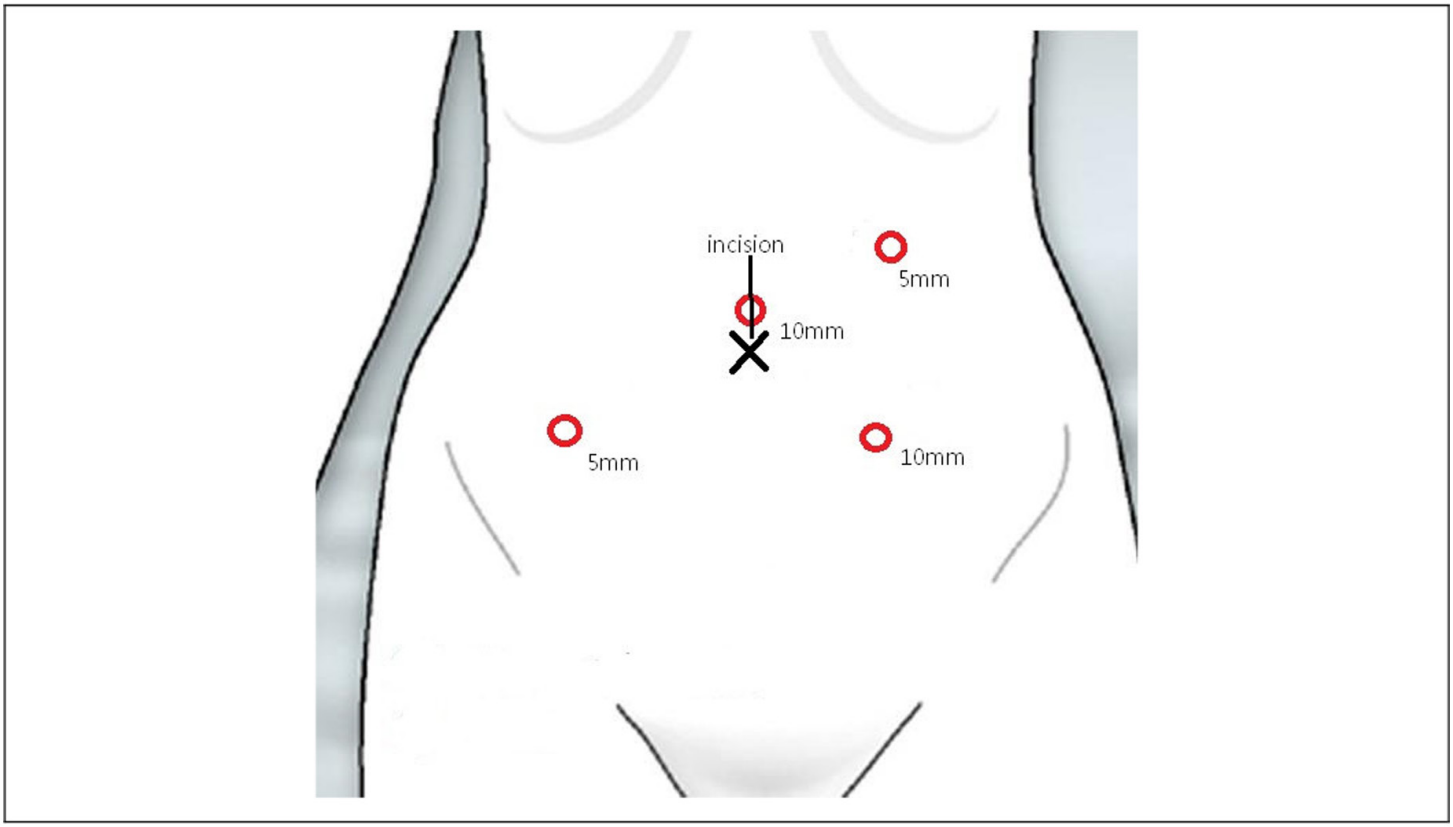
Laparoscopic Trocar Placement: The laparoscopic procedure involved the strategic placement of trocars: A primary 10‐mm port was inserted above the umbilicus, serving the dual purpose of establishing pneumoperitoneum and accommodating a 30° laparoscope. Three additional working ports were situated as follows: a 10‐mm port in the left iliac fossa, a 5‐mm port in the right iliac fossa, and another 5‐mm port in the left upper quadrant of the abdomen.

The planned surgical procedure entailed laparoscopic right hemicolectomy with subsequent ileocolic end‐to‐end anastomosis. The four trocars were positioned meticulously for optimal access and visualization. A primary 10‐mm port was strategically inserted above the umbilicus, establishing a pneumoperitoneum and facilitating a 30° laparoscope. Three additional working ports were placed: a 10‐mm port in the left iliac fossa, a 5‐mm port in the right iliac fossa, and another 5‐mm port in the left upper quadrant of the abdomen (see Figure 3 for precise details).

Mobilization of the affected area was meticulously performed using endoshears aided by a LigaSure device. Following complete mobilization of the ileocolic region, the affected bowel loop was exteriorized through a small suprapubic incision, and the involved segments were subsequently resected.

**FIGURE 4:**
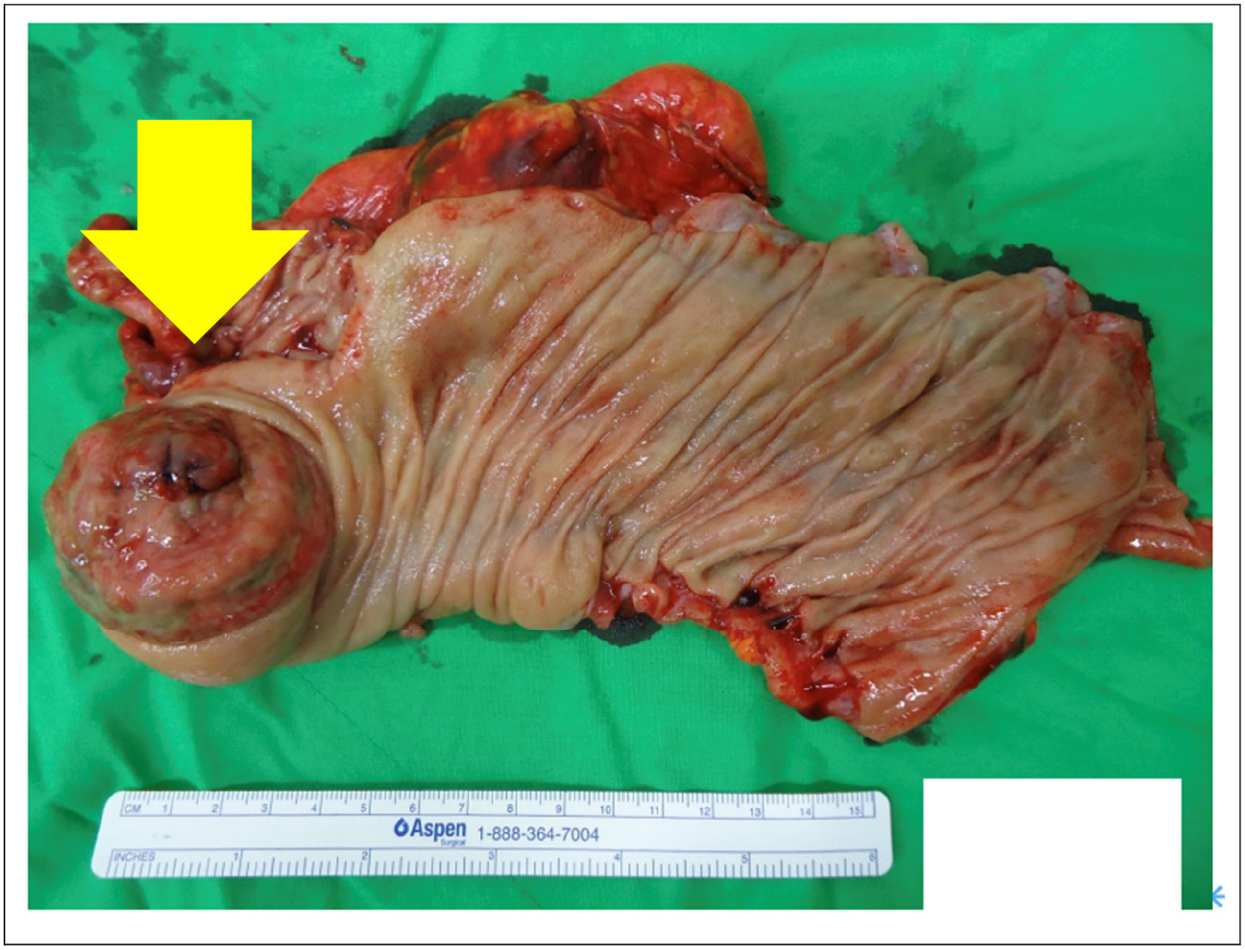
Specimen. Macroscopic examination: The examination of the specimen revealed a solitary exophytic polypoid tumor with dimensions measuring 6 × 5 × 2.5 cm. This tumor was located within the cecal region and was surrounded by pericolonic fat.

Macroscopic examination revealed an exophytic polypoid tumor measuring 6 × 5 × 2.5 cm, located within the cecal region and near the pericolonic fat (see Figure 4 for precise visual representations). Histological analysis confirmed the tumor as a DLBCL based on the observation of discohesive, large, and pleomorphic tumor cells that had infiltrated throughout the entire thickness of the colonic wall. Immunohistochemical assessment further substantiated the diagnosis, revealing positive staining for the Cluster of Differentiation 79 alpha (CD79a) and B‐cell lymphoma 2 (Bcl‐2) markers (see Table 1, delineating the specific functions and roles of CD79a and Bcl‐2, which are pivotal in the immunophenotypic characterization of lymphomas).

**TABLE 1:** Functions of immunohistochemical markers CD79a and Bcl‐2.

Marker	Function
CD79a	A component of the B‐cell receptor complex; used as a diagnostic marker in immunophenotyping to identify B‐cell lineage in lymphomas.
Bcl‐2	An anti‐apoptotic protein that regulates cell death (apoptosis); overexpression can contribute to oncogenesis and is associated with resistance to cancer therapies.

Abbreviations: Bcl‐2, B‐cell lymphoma 2; CD79a, Cluster of Differentiation 79 alpha.

The confirmed diagnosis was DLBCL, and tumor cells were also detected in three regional lymph nodes excised from the mesentery.

Postoperative recovery proceeded without significant complications, and the patient was discharged on the seventh day after the surgical procedure. Subsequently, the patient was transferred to the hematology department to initiate further therapeutic interventions.

## 4 CONCLUSION AND RESULTS (OUTCOME AND FOLLOW‐UP)

Following her discharge, the patient completed six cycles of R‐CHOP therapy (Rituximab + Cyclophosphamide + Doxorubicin + Vincristine + Prednisone), as advised by the hematologist, with treatments administered every 3 weeks over a period of 18 weeks. Up to the present date, she has not reported any side effects. She was also advised to schedule follow‐up visits to the outpatient department every 6 months for tumor monitoring. These follow‐up visits generally consist of evaluating symptom evaluation and a physical examination, together with a complete blood count and a biochemical profile including serum levels of lactate dehydrogenase (LDH). To date, there has been no signal of recurrence.

## 5 DISCUSSION

This report aimed to discuss two salient issues: the approach to adult ileocolic intussusception and the management of Gl lymphoma. Adult intussusception, characterized by the invasion of one part of the intestine into another, typically manifests in two forms within the ileocolic region: ileocolic intussusception, where the ileocecal valve acts as the lead point, and ileocecal intussusception, involving the prolapse of the ileum through the ileocecal valve.^5^ The ileocecal variant occurs 1.5 times more frequently than the ileocolic type.^5^, ^6^, ^7^ The literature provides limited comparative analyses of ileocecal and ileocolic intussusceptions. However, these two categories share overlapping diagnostic profiles. Clinically, adult intussusception often presents insidiously, manifesting as non‐specific symptoms resembling intestinal obstruction. Abdominal pain is the most commonly reported symptom, and nausea and vomiting are frequently observed.^8^ Notably, pain associated with adult intussusception typically presents periodically and intermittently, potentially obscuring the diagnosis and resulting in delayed treatment. Current data indicate that only approximately half of adult intussusception cases are diagnosed preoperatively.^9^ Upon physical examination, a palpable abdominal mass may be detected in approximately 24%–42% of patients with intussusception.^7^, ^8^ In cases of adult intussusception, laboratory tests typically yield non‐specific findings, even with bowel perforation or compromise. Plain abdominal radiography is often employed as a preliminary diagnostic tool to provide information regarding the existence and positioning of intestinal obstruction.^10^ In CT imaging, intussusception characteristically presents as an initial “target mass” appearance, displaying concentric rings with central areas of low attenuation. Progressive intussusception may be a multilayered phenomenon because of venous congestion and compressive forces exerted longitudinally on the intussusceptum.^11^ CT of the abdomen is the most reliable method for diagnosing intestinal intussusception, demonstrating superior efficacy to contrast radiography, ultrasonography, and colonoscopy.^11^, ^12^

In both ileocolic and ileocecal intussusceptions, a lead point is commonly identified,^13^ with a significant proportion attributed to malignant etiologies.^14^ In adult intussusception, 90% of cases exhibit identifiable lead points, usually signifying an underlying pathology. Both ileocolic and ileocecal intussusceptions predominantly involve benign etiologies, including benign tumors, lipomas, inflammatory conditions, Meckel's diverticulum, postoperative adhesions, and intussuscepted tubes. However, malignant causes account for approximately 65% of colon intussusceptions.^3^, ^14^

Surgical intervention is imperative in both ileocolic and ileocecal adult intussusception types. Furthermore, surgical management is advocated as the primary treatment option for all instances of colonic intussusception.^4^ This recommendation is underpinned by the high prevalence of malignancy, accounting for up to two‐thirds of cases. Common malignant etiologies include adenocarcinoma, leiomyosarcoma, lymphoma, and metastatic neoplasms.^3^, ^4^, ^10^, ^14^ Considering the increased risk of malignancy in adults with intussusceptions, surgical resection is typically advised. It has been proposed that when malignancy is suspected, reduction should not be attempted if the bowel appears inflamed, friable, or demonstrates signs of ischemia.^3^ Contemporary scholarship endorses surgical resection without prior reduction of adult intussusceptions, emphasizing that approximately half of both colonic and enteric intussusceptions are of malignant etiology.^8^ The management of intussusception attributed to malignancy involves considerable risks, including intraluminal seeding, venous embolization from ulcerated mucosal segments, anastomotic complications owing to tissue fragility from the reduction process, and an increased risk of bowel perforation.^4^, ^8^, ^12^, ^14^ Some case reports have described the effective management of benign ileocolic intussusception through laparoscopic intervention^15^ and mentioned that flexible colonoscopy is an essential diagnostic technique, especially for subacute or chronic large bowel obstructions secondary to intussusception. Flexible colonoscopy facilitates the diagnosis and determination of the location of the intussusception, and allows for the detection of any intrinsic organic lesions acting as lead points. Although this may offer therapeutic opportunities, the risk of tumor seeding owing to this intervention has not been well established in the literature.^3^

Here, we report a case of large B‐cell lymphoma, a subtype of GI lymphoma. The GI tract is recognized as the predominant site of extranodal lymphoma manifestation.^16^ Most studies indicate that the stomach is the site most frequently affected by GI lymphoma, accounting for 50%–60% of cases. The small intestine follows in terms of incidence, while the colon, rectum, and esophagus are less commonly involved, with each one involving less than 1% of cases.^17^, ^18^, ^19^ Gl lymphomas represent a varied group, accounting for 5%–20% of all non‐Hodgkin lymphomas and 30%–40% of extranodal lymphomas.^17^

Furthermore, GI lymphomas account for 1%–10% of all Gl malignancies.^20^ DLBCL represent the majority of these cases, with a significant portion also comprising non‐Hodgkin and mucosa‐associated lymphoid tissue (MALT) lymphomas. Also, DLBCL is known for its heterogeneity, and is an infrequent cause of acute obstructive symptoms and intussusception.^17^, ^21^ Nevertheless, aggressive Gl tumors require prompt identification and intervention to optimize patient outcomes and prevent disease progression.^17^, ^18^ The Dawson criteria are the quintessential diagnostic benchmarks for GI lymphoma, providing a systematic approach for accurately identifying the condition.^22^ Dawson et al. delineated the criteria for diagnosing primary GI lymphoma as follows: (1) absence of peripheral lymphadenopathy at presentation, (2) no mediastinal lymph node enlargement, (3) normal total and differential leukocyte counts, (4) bowel lesion predominance at laparotomy with regional lymph nodes affected, and (5) no hepatic or splenic lymphomatous involvement. Additionally, Ann Arbor staging with Musshoff modification is used for staging GI lymphoma, while the International Prognostic Index (IPI) determines the prognostic subgroups. However, both of these staging systems are overly complex and can be more confusing than beneficial, as they document more features of lymphoma than is necessary.^23^ The Paris Staging System, a well‐known TNM system, has become increasingly relevant for GI lymphoma.^19^ This staging system adequately records: (1) depth of tumor infiltration, (2) extent of nodal involvement, and (3) specific lymphoma spreading. It is adjusted to the GI origin of the lymphoma and considers the histopathological characteristics of extranodal B and T cell lymphomas. Using this system in future studies will allow for accurate comparison of reported cohorts and rapid accumulation of good data for proper stratification of patients in terms of risk and treatment options.^23^

In GI lymphomas, precise diagnosis and staging are crucial for formulating an effective treatment strategy. Pretreatment evaluations employ an array of diagnostic tools, including endoscopic ultrasound, endoscopic biopsy, CT, magnetic resonance imaging, 18F‐fluorodeoxyglucose positron emission tomography (PET), and molecular markers to establish the extent and nature of the disease.^24^, ^25^ In managing lymphomas, contrast‐enhanced modalities and functional imaging, such as perfusion CT, are pivotal in treatment monitoring and response assessment. Emerging technologies such as hybrid PET‐CT imaging and novel PET tracers, including 18F‐fluoro‐thymidine, have emerged as significant advancements, offering refined diagnostic and prognostic capabilities.^26^

In the last two decades, significant advancements have occurred in immunohistochemical profiling for the diagnosis, staging, and treatment of GI lymphomas. Key developments include the use of monoclonal antibodies such as rituximab and novel therapies targeting specific cell surface antigens. Progress in molecular understanding has led to new treatments such as anti‐CD20, CD22, CD30, CD40, and anti‐VEGF agents.^27^, ^28^ Recent research has highlighted the importance of combining clinical and morphological data with key immunohistochemical markers for prognostic assessment of primary GI lymphoma. The proliferation marker Ki‐67, the apoptosis regulator bcl‐2, and the tumor suppressor p53 are particularly significant. A high Ki‐67 index indicates aggressive tumor behavior and a poor prognosis. Abnormal levels of bcl‐2 and p53 levels are associated with poor clinical outcomes, making them essential for the prognosis. The data revealed that Ki‐67 was highly expressed in 68.6% of cases, bcl‐2 in 32.8%, and p53 in 26.2%, with high Ki‐67 and p53 levels particularly associated with more aggressive DLBCL, indicating potentially poorer outcomes.^29^

Currently, up to 60% of DLBCL patients achieve cure with first‐line immunochemotherapy regimens such as R‐CHOP, which combines cyclophosphamide, doxorubicin, vincristine, prednisone, and rituximab.^30^ Despite this success, the heterogeneity of DLBCL hinders the application of novel targeted therapies. Recent years have seen frequent discussions on molecular subgroups of DLBCL. Pioneering work by Alizadeh et al. utilized DNA microarrays to categorize DLBCLs based on gene expression patterns similar to either normal germinal center B cells (GCB) or activated peripheral B cells (ABC), leading to a “cell‐of‐origin” classification into GCB and ABC subtypes.^31^ Subsequent molecular profiling studies have suggested alternative classification systems.^32^, ^33^, ^34^ Morin et al. examined these studies and noted varying survival rates post‐R‐CHOP treatment across molecular subtypes.^35^ While R‐CHOP remains the gold standard treatment for DLBCL,^36^ ongoing research promises the development of novel, more precise therapies in the future.

This case report describes the case of a 71‐year‐old female with ileocolic intussusception precipitated by primary cecal B‐cell lymphoma. The patient was appropriately managed by laparoscopic resection. Postoperative reassessment guided by Ann Arbor staging confirmed the diagnosis of GI large B‐cell lymphoma (Grade I), and immunohistochemical analysis revealed positive staining for the CD79a and Bcl‐2 markers. Notably, CD79a expression indicates its utility as an adjunct to CD20, potentially compensating for its diminished expression owing to the plasmacytic differentiation of lymphoma cells or prior rituximab therapy.^28^ Further clinical and prognostic evaluations revealed no significant findings.

To sum up, large B‐cell lymphoma, although not rare, is relatively uncommon in clinical practice. The preoperative diagnosis of large B‐cell lymphoma leading to intussusception in adults presents significant challenges owing to the manifestation of non‐specific symptoms. Surgical intervention is often necessary to definitively identify the underlying etiology. Although colonoscopy is a valuable diagnostic tool, it is not a viable treatment option for this condition.

GI lymphoma predominantly occurs in the gastric region, with large B‐cell lymphoma being the most prevalent type. Staging criteria, including the Dawson criteria, Ann Arbor staging with Musshoff modification, and Paris staging, can be used for GI lymphomas. Emerging diagnostic methods such as hybrid PET/CT imaging and novel PET tracers have proven valuable in diagnosing GI lymphoma. Additionally, we present the latest immunohistochemical methods for diagnosing GI lymphoma.

## AUTHOR CONTRIBUTIONS

**Hao‐Cheng Chang:** Writing – original draft. **Jung‐Cheng Kang:** Supervision. **Ta‐Wei Pu:** Conceptualization; writing – review and editing. **Ruei‐Yu Su:** Resources. **Chao‐Young Chen:** Supervision. **Je‐Ming Hu:** Supervision; writing – review and editing.

## CONFLICT OF INTEREST STATEMENT

The authors have no conflict of interest to declare.

## CONSENT

Written informed consent was obtained from the patient to publish this report.

## Data Availability

The data that support the findings of this study are available on request from the corresponding author. The data are not publicly available due to privacy or ethical restrictions.
